# Autosomal Dominant Pseudohypoaldosteronism Type 1 in an Infant with Salt Wasting Crisis Associated with Urinary Tract Infection and Obstructive Uropathy

**DOI:** 10.1155/2013/524647

**Published:** 2013-12-19

**Authors:** Sasigarn A. Bowden, Corin Cozzi, Scott E. Hickey, Devon Lamb Thrush, Caroline Astbury, Sushma Nuthakki

**Affiliations:** ^1^Division of Endocrinology, Nationwide Children's Hospital/The Ohio State University College of Medicine, Columbus, OH 43205, USA; ^2^Division of Neonatology, Nationwide Children's Hospital/The Ohio State University College of Medicine, Columbus, OH 43205, USA; ^3^Division of Molecular & Human Genetics, Department of Pediatrics, Nationwide Children's Hospital/The Ohio State University College of Medicine, Columbus, OH 43205, USA; ^4^Department of Pathology and Laboratory Medicine, Nationwide Children's Hospital/The Ohio State University College of Medicine, Columbus, OH 43205, USA

## Abstract

Type 1 pseudohypoaldosteronism (PHA1) is a salt wasting syndrome caused by renal resistance to aldosterone. Primary renal PHA1 or autosomal dominant PHA1 is caused by mutations in mineralocorticoids receptor gene (*NR3C2*), while secondary PHA1 is frequently associated with urinary tract infection (UTI) and/or urinary tract malformations (UTM). We report a 14-day-old male infant presenting with severe hyperkalemia, hyponatremic dehydration, metabolic acidosis, and markedly elevated serum aldosterone level, initially thought to have secondary PHA1 due to the associated UTI and posterior urethral valves. His serum aldosterone remained elevated at 5 months of age, despite resolution of salt wasting symptoms. Chromosomal microarray analysis revealed a deletion of exons 3–5 in *NR3C2* in the patient and his asymptomatic mother who also had elevated serum aldosterone level, confirming that he had primary or autosomal dominant PHA1. Our case raises the possibility that some patients with secondary PHA1 attributed to UTI and/or UTM may instead have primary autosomal dominant PHA1, for which genetic testing should be considered to identify the cause, determine future recurrence risk, and possibly prevent the life-threatening salt wasting in a subsequent family member. Future clinical research is needed to investigate the potential overlapping between secondary PHA1 and primary autosomal dominant PHA1.

## 1. Introduction

Pseudohypoaldosteronism (PHA) is a disorder caused by aldosterone resistance with subsequent impaired sodium reabsorption and potassium excretion. The broad category of PHA includes PHA type 1 (PHA1) and PHA type 2 (PHA2, also known as Gordon's syndrome or familial hyperkalemic hypertension). PHA1 is subdivided into primary (genetic) and secondary (or transient) forms. Primary PHA1 has two clinically and genetically distinct forms [1]: (1) the autosomal dominant or sporadic form (also called renal form), caused by mutations in the mineralocorticoid receptor (MR) coding gene *NR3C2* [2] and (2) the autosomal recessive or generalized PHA1, which is caused by mutations in genes encoding subunits of the epithelial sodium channel [3]. The secondary (transient) PHA1 has been described in infants suffering from urinary tract malformations or urinary tract infections (UTI) or both [4–12]. We present the first case report of autosomal dominant PHA1 with an intragenic deletion of *NR3C2*, presenting with salt wasting crisis associated with UTI and posterior urethral valves, thereby mimicking secondary PHA1.

## 2. Case Presentation

**Figure 1:**
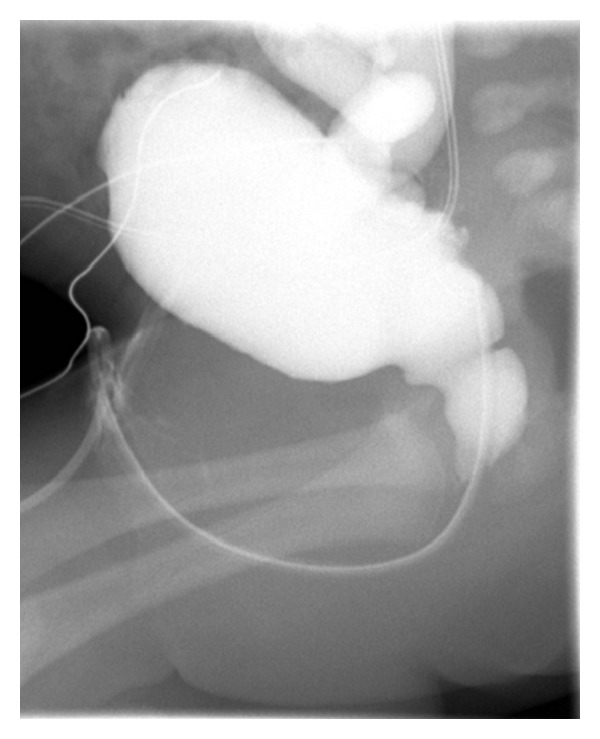
Voiding cystourethrogram demonstrating a thick-walled, trabeculated bladder with an enlarged posterior urethra that is consistent with posterior urethral valves.

The male patient, the second child of nonrelated Caucasian parents, was born at term after an uneventful pregnancy with a birth weight of 3,800 grams. Family history was significant for two maternal great uncles with early infancy deaths of unknown cause. He presented to the emergency department at 14 days of age with a one-week history of poor feeding, vomiting, lethargy, and failure to thrive, with a 20% weight loss since birth. An intraosseous line was placed due to difficulty obtaining intravenous access for volume resuscitation. Endotracheal intubation was also performed. His initial blood chemistries showed hyponatremia, marked hyperkalemia, metabolic acidosis, and elevated BUN and creatinine (Table 1). Complete blood count was remarkable for leukocytosis, bandemia, and thrombocytopenia (platelet count of 18,000). Cardiac monitoring demonstrated widened QRS complexes and peaked T waves. Acute management of symptomatic hyperkalemia included sodium polystyrene sulfonate, calcium chloride, sodium bicarbonate, insulin, and dextrose containing intravenous fluids. He was given a stress dose of hydrocortisone for a presumptive diagnosis of congenital adrenal hyperplasia and an empiric antibiotic therapy for UTI as his urinalysis showed pyuria. Urine culture revealed >100,000 colonies/cc of *Escherichia coli*. His blood and cerebrospinal fluid cultures were normal. Renal ultrasound showed moderate ureteropelvicaliectasis with debris in the ureters, collecting system, and bladder. Voiding cystourethrography (Figure 1) demonstrated posterior urethral valves and grade IV vesicoureteral reflux. Hydrocortisone was discontinued when the 17-hydroxy progesterone level was normal. Metabolic acidosis improved with normalized serum sodium and potassium levels within a few days of intravenous fluids and antibiotic therapy. Serum aldosterone obtained at 26 days of age was elevated (Table 1). His renal function improved after corrective surgery with cystoscopic transurethral resection of posterior urethral valves performed at 20 days of age. He continued to have feeding difficulty, stridor, and cough after each feeding, prompting placement of a gastrostomy tube. Otolaryngology evaluation revealed a submucous cleft palate and bifid uvula. At 5 months, his repeat serum aldosterone remained elevated while plasma renin activity was normal. He had good growth and weight gain with his nutrition mostly given through gastrostomy tube. He maintained normal electrolytes without salt supplementation.

**Figure 2:**
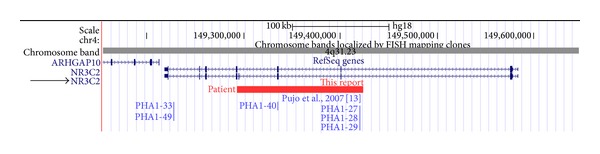
Schematic of a deletion spanning exons 3–5 in our patient (as shown in red bar) compared to other single exons deletions detected by quantitative real-time PCR using exon-spanning primers (as shown in blue vertical line) in exon 3 (families PHA1-27, PHA1-28, and PHA1-29), exon 4 (family PHA1-40), and exon 8 (families PHA1-33 and PHA1-49), as reported by Pujo et al. in 2007 [13]. Deletion map corresponds to *NR3C2 *isoform 1 (NM_000901.4), as indicated by black arrow.

Chromosomal microarray was obtained due to the history of poor feeding of unidentified etiology in a full-term infant. The results of the array revealed a 130 kb loss within chromosome band 4q31.23 [arr 4q31.23(149,293,435–149,423,596)×1], which deletes exons 3 through 5, and the surrounding intronic regions of the major isoform of *NR3C2* (Figure 2).

Simultaneous microarray analysis on the patient's mother showed the same deletion in 4q31.23, confirming that the mutation was maternally inherited. The mother had no history of salt losing syndrome during infancy and had never been hospitalized for any illnesses. She reported a history of salt craving during childhood until 15 years of age. She had elevated serum aldosterone level but normal plasma renin activity.

### 2.1. Methods of Genetic Testing

Microarray-based comparative genomic hybridization was performed using a custom-designed 135K-feature oligonucleotide array (Roche NimbleGen, Madison, WI, USA). The array was designed for the identification of DNA copy number gains and losses associated with chromosomal imbalances and will detect aneuploidy, deletions, and duplications of the loci represented on the array. It will not detect balanced rearrangements including inversions, reciprocal translocations, Robertsonian translocations, and insertions. The probes on the array have an average spacing of one probe every 35 kb throughout the genome and one probe every 10 kb in regions known to have clinical significance. The labeling, hybridization, and posthybridization washing stages were performed using NimbleGen's protocol with minor modifications. Nucleotide locations are based on UCSC Genome Browser (Build hg18, March 2006).

## 3. Discussion

Severe hyponatremic dehydration with hyperkalemia and metabolic acidosis is a life-threatening condition in infancy that requires prompt treatment to prevent death. Our patient presented with salt wasting crisis with marked hyperkalemia and abnormal electrocardiogram requiring several pharmacologic interventions and aggressive fluid therapy. Adrenal conditions, particularly congenital adrenal hyperplasia, are high on the differential diagnosis. Other possible causes include congenital adrenal hypoplasia, isolated aldosterone deficiency, or pseudohypoaldosteronism. Our investigation revealed that our patient had a UTI which led to the diagnosis of underlying obstructive uropathy due to posterior urethral valves. Endocrinologic evaluation demonstrated an elevated aldosterone level in the setting of hyponatremia and hyperkalemia, consistent with PHA1.

Patients with primary (genetic) PHA1 generally present in the neonatal period with renal salt wasting and failure to thrive. The generalized or autosomal recessive PHA1 is caused by loss-of-function mutations in the epithelial sodium channel present in many organs [3]. Patients with generalized PHA1 have a more severe course with salt wasting from kidney, colon, sweat, and salivary glands and require massive sodium supplementation throughout life. In contrast, the renal or autosomal dominant PHA1 (AD-PHA1) is caused by mutations in the *NR3C2* gene which codes for the MR, with a phenotypic expression restricted to the kidney [13, 14]. Clinical symptoms usually remit with age and salt supplementation is generally not required after the age of 2 years as patients compensate for their defective mineralocorticoid receptors by upregulating the renin-angiotensin system [13] or by increasing dietary salt intake and maturation of sodium reabsorption function of the renal tubules with increasing age [2]. Although less severe in its course, AD-PHA1 has been reported to be associated with high infant mortality rate [15].

The secondary (transient) form of PHA1 is due to temporary aldosterone resistance and has been described in over 100 young infants in association with UTI and/or urinary tract malformations (UTM) of any kinds such as ureterohydronephrosis, ureterocele, ureteropelvic junction obstruction, or posterior urethral valves [4–12]. Ninety percent of patients are less than 3 months of age and suffer from UTM, with associated UTI in 89% of them [12]. Approximately 10% of patients have UTM in the absence of UTI or isolated UTI. In the majority of reported patients, treatment of UTI and UTM reversed the electrolyte abnormalities, but, in some patients, salt supplementation was necessary for weeks or months [10], and some had persistently elevated serum aldosterone levels [16]. Unlike the primary forms, the underlying pathogenesis of secondary PHA1 is unclear. Postulated possible mechanisms are parenchymal scarring secondary to obstruction and tubular aldosterone resistance secondary to endotoxin damage of the aldosterone receptors from cytokine factors such as transforming growth factor-*β* (TGF-*β*) [9]. TGF-*β* is known to decrease MR sensibility. Furthermore, the severity of salt wasting is inversely correlated with age, supporting a role for tubular immaturity [10]. Many authors conclude that urine culture and renal ultrasound/imaging studies are essential to differentiate secondary PHA1 from the genetic form [10–12]. Genetic analysis was not performed in any reported cases with secondary PHA1 due to their transient course.

Our patient, initially thought to have secondary PHA1 due to associated UTI and UTM, in fact, has a primary AD-PHA1, as he had a large deletion in the *NR3C2 *gene detected by microarray analysis. No other cases with AD-PHA1 have been reported in which the diagnosis was ascertained by chromosomal microarray. The patient's deletion involves exons 3 through 5 which would be expected to disrupt both the highly conserved DNA-binding domain and the ligand-binding domain of the MR protein [17]. The patient has persistent elevated serum aldosterone, even when asymptomatic, consistent with previous descriptions that individuals with AD-PHA1 can have lifelong elevated aldosterone levels, while plasma renin decreases to normal levels with increasing age [1]. His mother has the same gene deletion, which confirms autosomal dominance in this family. The elevated serum aldosterone level in the mother is consistent with what has been described in asymptomatic affected family members, providing further evidence for variable expressivity in which some individuals with the deletion have a subclinical course. It is possible that the mother's two uncles who died in early infancy could have had AD-PHA1, as a number of unexplained infant deaths have been reported in families with AD-PHA1 [15]. This underscores the need for identification of AD-PHA1 in a kindred to provide genetic counseling and screening for salt wasting in offspring born to affected individuals to prevent the life-threatening salt wasting crisis.

It is unknown whether the UTI and UTM in this patient are associated with his *NR3C2* gene mutation or merely a coincidence that occurs as a precipitating factor for his salt wasting crisis. There has been one case report of an infant diagnosed with secondary PHA1 during the course of pyelonephritis and later found to have AD-PHA1. While *NR3C2* DNA sequencing was not performed in this patient, his family studies showed elevated aldosterone and renin levels in his mother and two siblings, documenting autosomal dominant PHA1 [18]. This patient and our patient were the two cases in the literature to date that were diagnosed with AD-PHA1 after urologic manifestations mimicking secondary PHA1, suggesting that there could be additional cases presenting in a similar way. Since the association between secondary PHA1 and UTI/UTM has been described in over 100 cases in the literature, further investigations to elucidate the potential underlying genetic basis are warranted. Only if more cases are identified similar to our patient, more insights into pathogenesis as well as genotype-phenotype correlations in renal PHA1 shall be gained.

## 4. Conclusion

This is the first case report of autosomal dominant PHA1 with an intragenic deletion of *NR3C2* in an infant initially thought to have secondary PHA1 associated with UTI and posterior urethral valves. Our case challenges the current pathophysiologic paradigm of secondary PHA1, raising the possibility that some patients with secondary PHA1, especially those presenting with UTI and UTM, may in fact have primary AD-PHA1. As primary AD-PHA1 has implications for future recurrence risk in the family with potential life-threatening salt wasting, it is crucial to perform genotype investigation in patients with secondary PHA1 associated with UTM and/or UTI to identify primary PHA1. Repeat measurement of serum aldosterone can be used as a screening tool; if elevated after UTM and UTI are treated, then *NR3C2* DNA testing, including deletion analysis, should be considered. Larger case series investigating the possible genetic basis in secondary PHA1, which could possibly be overlapping with primary renal PHA1, would be of great benefit to gain more insights into disease pathogenesis.

## Figures and Tables

**Table 1:** The biochemical data of the patient.

	On admission	Day 2	After surgery	Followup
Age	14 days	15 days	3 weeks	5 months
Serum sodium (132–142 mmol/L)	126	141	135–138	141
Serum potassium (4–6.4 mmol/L)	8.2	4.2	4.4–5.5	4.8
Serum bicarbonate (18–27 mmol/L)	13	22	22–24	25
Serum BUN (4–15 mg/dL)	81	77	5–10	9
Serum creatinine (0.1–0.3 mg/dL)	1.88	1.94	0.35–0.36	0.4
Urine FENa (%)	10.94%			0.47%
Serum aldosterone (5–90 ng/dL)	Quantity insufficient		1000	190
PRA (235–3700 ng/dL/hr)				1533

FENa: fractional excretion of sodium; PRA: plasma renin activity.

## Abbreviations

AD-PHA1: Autosomal dominant pseudohypoaldosteronism type 1
MR: Mineralocorticoid receptor
PHA1: Pseudohypoaldosteronism type 1
UTI: Urinary tract infection
UTM: Urinary tract malformations.
